# Principles and Standards for Designing and Managing Integrable and Interoperable Transformed Health Ecosystems

**DOI:** 10.3390/jpm13111579

**Published:** 2023-11-04

**Authors:** Bernd Blobel, Pekka Ruotsalainen, Frank Oemig, Mauro Giacomini, Pier Angelo Sottile, Frederik Endsleff

**Affiliations:** 1 Medical Faculty, University of Regensburg, 93053 Regensburg, Germany; 2 Faculty European Campus Rottal-Inn, Deggendorf Institute of Technology, 94469 Deggendorf, Germany; 3 First Medical Faculty, Charles University Prague, 11000 Staré Mĕsto, Czech Republic; 4 Faculty of Information Technology and Communication Sciences, Tampere University, 33100 Tampere, Finland; pekka.ruotsalainen@tuni.fi; 5 IT-Consulting in Healthcare, 45472 Mülheim, Germany; frank@oemig.de; 6 Department of Informatics, Bioengineering, Robotics and System Engineering, University of Genoa, 16145 Genoa, Italy; mauro.giacomini@dibris.unige.it; 7 BI Health Srl, 00185 Rome, Italy; p.sottile@bihealth.it; 8 IT Architecture, Centre for IT and Medical Technology (CIMT), The Capital Region of Denmark, 2100 Copenhagen, Denmark; frederik.endsleff@regionh.dk

**Keywords:** 5P medicine, ecosystem, system architecture, knowledge representation, knowledge management, modeling, integration, interoperability

## Abstract

The advancement of sciences and technologies, economic challenges, increasing expectations, and consumerism result in a radical transformation of health and social care around the globe, characterized by foundational organizational, methodological, and technological paradigm changes. The transformation of the health and social care ecosystems aims at ubiquitously providing personalized, preventive, predictive, participative precision (5P) medicine, considering and understanding the individual’s health status in a comprehensive context from the elementary particle up to society. For designing and implementing such advanced ecosystems, an understanding and correct representation of the structure, function, and relations of their components is inevitable, thereby including the perspectives, principles, and methodologies of all included disciplines. To guarantee consistent and conformant processes and outcomes, the specifications and principles must be based on international standards. A core standard for representing transformed health ecosystems and managing the integration and interoperability of systems, components, specifications, and artifacts is ISO 23903:2021, therefore playing a central role in this publication. Consequently, ISO/TC 215 and CEN/TC 251, both representing the international standardization on health informatics, declared the deployment of ISO 23903:2021 mandatory for all their projects and standards addressing more than one domain. The paper summarizes and concludes the first author’s leading engagement in the evolution of pHealth in Europe and beyond over the last 15 years, discussing the concepts, principles, and standards for designing, implementing, and managing 5P medicine ecosystems. It not only introduces the theoretical foundations of the approach but also exemplifies its deployment in practical projects and solutions regarding interoperability and integration in multi-domain ecosystems. The presented approach enables comprehensive and consistent integration of and interoperability between domains, systems, related actors, specifications, standards, and solutions. That way, it should help overcome the problems and limitations of data-centric approaches, which still dominate projects and products nowadays, and replace them with knowledge-centric, comprehensive, and consistent ones.

## 1. Introduction

The paper at hand is a revised and extended version of the pHealth 2022 Keynote paper, published in [1]. It addresses the globally ongoing transformation of health and social care systems and provides a model and framework for representing and managing them. To realize sustainable and compatible health ecosystems, their design, implementation, and management must follow internationally accepted principles and standards. The authors introduce a theoretical approach, common principles, standards, and practical solutions for designing and managing integrable and interoperable health ecosystems that are proven through some practical demonstrations. The described solution has also been successfully applied to and integrated into the standards from ISO, CEN, IEEE, and other standardization organizations. The paper explains those principles and how to navigate the related standards jungle.

An ecosystem is a system or network of living and nonliving interconnecting and interacting elements to meet specific objectives [2]. The transformation aims at mastering challenges such as the ongoing demographic changes towards aging, multi-diseased societies, the related development of human resources, health and social services consumerism, medical and biomedical progress, and exploding costs for health-related R&D as well as health services delivery [3]. An overview of requirements and solutions for managing healthcare transformation towards 5P medicine can be found in [4] or [5]. A detailed description of the architectural approach is available in [6]. The transformation is bound to fundamental organizational, methodological, and technological paradigm changes [7]. Thereby, the care type advances from empirical or phenomenological medicine through evidence-based medicine, person-centered medicine, personalized medicine, 5P medicine, and ubiquitous personal health. Organizationally, the systems turn from organization-centered local services through cross-organizational local services and distributed local and remote services to ubiquitous care. Regulated professionals manage the first three organizational settings, while for the other three regulated and non-regulated professionals, non-professionals such as the subject of care and his/her relations and technical systems play the role of actors. In the phenomenological medicine care type, domain-specific general services are provided to humanity as one solution fits all. In evidence-based medicine, domain-specific services are provided to disease-specifically defined groups. In person-centered medicine, individuals are served with multiple domains’ disease-specifically interrelated services, including telemedicine. Personalized medicine provides multiple domains’ services to the individual’s personal disposition. Systems medicine—also called 5P medicine, i.e., personalized, preventive, predictive, participative precision medicine—provides integrated cross-domain services to the individual in personal, environmental, social, occupational, and behavioral contexts, thereby deploying life sciences, social sciences, and engineering sciences, as well as specialties such as the bunch of omics disciplines and others. Ubiquitous personal health serves the individual under comprehensive focus with integrated services. From a methodological perspective, empirical medicine practices are based more or less on objectivized observations, justified with pattern recognition or experiences. Evidence-based medicine advances observations through objective evaluations, statistically justified with group-specific treatment outcomes stored in records, registries, etc. Person-centered medicine is realized as managed care, leading the subject of care through the care process and justifying the process through process management and best medical practice guidelines. A big advancement is provided through personalized medicine based on the pathology of the individual disease, clinically justified with the individual’s status and context. Systems medicine understands the detailed pathology based on multiple domains, scientifically justified through individual status and context. Ubiquitous personal health provides services dynamically tailored for the subject of care, anywhere and anytime. The methodological paradigm changes are accompanied by transformations regarding the representation style of the practice outcome. Phenomenological medicine represents the observations as data stored in local data repositories. As evidence-based medicine contains data from multiple sources stored in central data repositories, the meaning of the data must be justified and verified against the source’s intent, leading to information. For representing the outcome of person-centered medicine, agreed-upon terminologies deployed in the Disease Management Program (DMP) Best Practice Guidelines are used, representing a cross-organizational business process. Personalized medicine requires the representation of disciplinary concepts in the situational context in the sense of knowledge representation and management. Systems medicine is represented through multi-disciplinary concepts in a comprehensive context, requiring knowledge space management. The different care types and related representation styles require different standards to manage them. Those standards range from data standards through information modeling standards, terminology, and process standards up to domain ontology standards, and for systems medicine, finally, top-levelontology standards guiding the management of multiple ontologies.

Interoperability advances thereby from signal sharing through data sharing, information sharing, knowledge sharing at the IT concept level, knowledge sharing at the business concept level, knowledge sharing at the domain level (cross-domain cooperation), up to skills-based knowledge sharing (moderated end-user collaboration). Such transformation must be supported using appropriate technologies from mobile devices through wearable and implantable sensors and actuators, pervasive sensors, actuators, and network connectivity, up to the micro, molecular, and quantum levels. By combining the advancements in societies, sciences, including data sciences, and technologies, health and social care systems are transformed into 5P medicine ecosystems. The outcome of the process enables early identification, proactive intervention, and a full understanding of the course of disease, i.e., its pathology and its effective treatment. It allows for health service provision everywhere, anytime, thereby individualizing the system according to the status, context, needs, expectations, wishes, etc., of the subject of health and social care. More details can be found at [1]. Table 1 summarizes the organizational, methodological, technological, and standardization paradigm changes in transformed health and social care ecosystems.

Table 1 clarifies that the advancement in health and social care paradigms must be accompanied by related advancements in the standard world. Healthcare transformation must be supported through appropriate technologies. The “Standards” column just addresses minimal needs for the representation and specification of real-world business systems and documents the increasing requirements. The design and implementation of information and communication technology (ICT) solutions require, of course, other standards and specifications, which are also discussed in this paper.

Table 2 presents the objectives of 5P medicine, the requirements (characteristics) for enabling those objectives, as well as the methodologies and technologies to realize them [8,9].

In the following, we will provide a comprehensive and scientifically sound representation of 5P medicine ecosystems as well as the standards for defining, modeling, and implementing the related system components.

## 2. Representation of 5P Medicine Ecosystems

**Figure 1:**
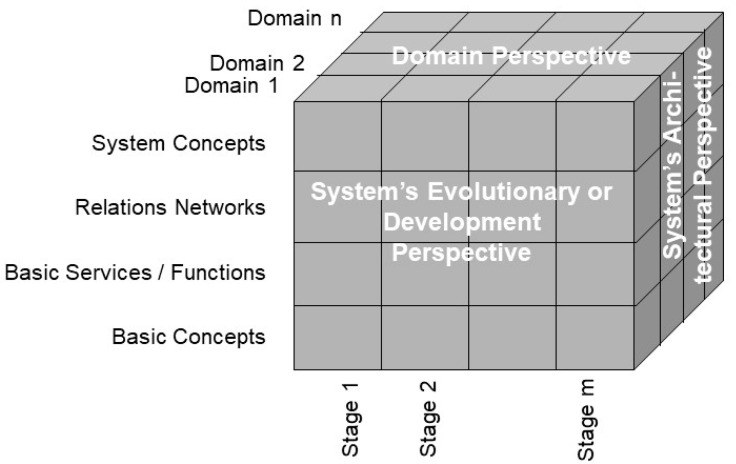
Generic model to represent ecosystems (after [10], changed).

To represent any system, we can deploy systems theory. The simplest way is the black-box methodology, which characterizes any living or non-living system’s coarse behavior and functionality through an input-output analysis. However, we cannot understand the functionality without considering the structure and functionality of the system components according to the white-box approach. Figure 1 represents an architectural system model by considering three aspects or dimensions:

- The system’s architectural perspective, representing the system’s composition/decomposition or specialization/generalization;
- The system’s domain perspective, representing the involved domains and their actors;
- The system’s evolutionary or development perspective.

Therefore, 5P medicine ecosystems must be structurally and functionally represented in a comprehensive way. For describing such ecosystems, universal type theory and universal logics, formally represented using the Barendregt Cube [11], can be deployed. Thereby, all domains involved, specific objectives and contexts, the system elements, their composition and decomposition, including internal and external relationships, and all steps in the development process represented as system views must be considered, thereby strictly following the good modeling best practices [12].

**Figure 2:**
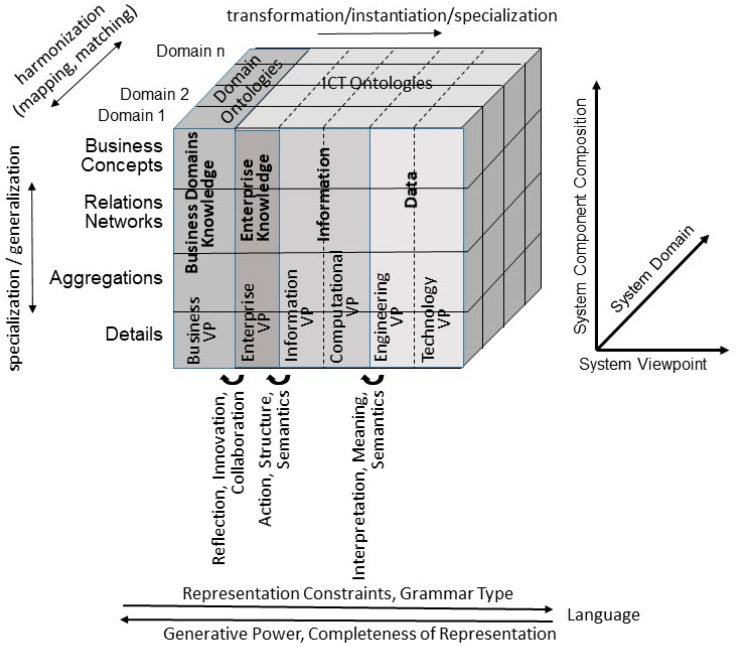
Model and framework for representing multi-domain, knowledge-based, ontology-based, policy-driven ecosystems [1].

P5 medicine requires the communication and cooperation of actors from multiple disciplines with specific perspectives, contexts, and objectives, using their special methodologies, languages, knowledge, and skills to name and define the business use case concepts and relations for correctly deriving the system requirements. The challenge of P5 medicine ecosystems is the proper representation, mapping, and matching of their domain-specific knowledge at any representation level. The knowledge spaces for the different viewpoints range from the business view through the enterprise view, the information view, and the computational view up to the technology view, as defined in ISO/IEC 10746 Open Distributed Processing [13]. The business view addresses the real-world business system. The enterprise view of the ICT system considers the management of the business process. The information view and the computational view deal with the semantic interpretation of data as information, while the engineering and technology views’ concerns are the implementable solution and its maintenance based on data. The business view is represented by domain ontologies, while the last five views are defined using corresponding ICT ontologies. For representing the different viewpoints, different presentation language types with increasing expressivity and increasing constraints according to the Chomsky grammar level are used. However, a highly expressive knowledge representation is less likely to properly consider context and implicit knowledge as being complete and decidable. Therefore, the limitations on data spaces and data interoperability are insufficient for correctly and consistently representing ecosystems. Consequently, for correctly defining relations for integration or interoperability in a more constraint view, we have always had to start representing the business system at the view with the highest generative power and transform thereafter the models up to the view to be managed. The language types start with domain-specific or natural languages to represent the business system, by domain experts. At the next level, business process modeling languages like BPML and BPML+ are used, followed by information representation languages such as vocabularies, thesauri, taxonomies, glossaries, data dictionaries, or information models, and finally data representation languages such as data/meta-data definitions, database management system (DBMS) schemes, or programming languages (see Table 3). In their data modeling hierarchy, Hoberman et al. [14] call the aforementioned representation levels as very high level, high level, logical level, and physical level, respectively. The corresponding representation of a multi-domain, ontology-based, policy-driven P5 ecosystem using the model and framework of the ISO 23903 Interoperability and Integration Reference Architecture [15], discussed in the next section, is shown in Figure 2.

The mapping between elements from different domains or different viewpoints can only be performed at the horizontal level, i.e., at the same level of granularity. To get there, components must be specialized or generalized, respectively.

For designing, developing, and implementing P5 medicine ecosystems, we must generically model the system architecture and the unified process around it. Thereafter, we have to formally represent the domains involved in the use case of the business system considered. Then, we have to represent the different views in the contexts and from the perspectives of the domain experts participating in the business use case. A domain controlling the business system behavior and therefore being relevant across all specific use cases is, e.g., the policy domain, covering procedural, legal, administrative, security, privacy, and trustworthiness, as well as ethical aspects.

For managing organizations to meet their objectives, interests, and needs, strategic, operational, and tactical aspects must be considered. In that context, related standards and procedures have to be established alongside policies to create a strong governance structure. Security and privacy policies address operational needs [16].

Consequently, we need architecture standards, knowledge representation, and management standards, including ontology standards and terminology standards, for all domains involved in the ecosystem. Furthermore, policy standards, business process modeling standards, information standards, and data standards to model and implement the 5P medicine ecosystem in a compliant and conformant way are necessary. In that context, we cannot ignore quality criteria standards to enable quality assessment (self-assessment and/or assessment by certified assessors) of pHealth digital tools such as IEC 82304-2:2021 Health Software-Part 2: Health and wellness apps-Quality and reliability [17]. Each standard family will be discussed and exemplified in some detail in the next sections.

## 3. Standards for Modeling 5P Medicine Ecosystems

The solution for designing, managing, and implementing the intended ecosystem is a system-theoretical white box, architecture-centric, ontology-based, and policy-controlled approach, meanwhile standardized as ISO 23903 Interoperability and Integration Reference Architecture–Model and Framework and re-used by many international Standards Developing Organizations (SDOs) such as ISO, CEN, IEC, IEEE, OMG, and HL7. Besides the definition of the modeling and system development process, ISO 23903 also covers challenges such as domain-specific knowledge representation and management at the epistemological level, as well as its harmonization. In that context, it supports not only the ontology development and harmonization but also the implementation of good modeling best practices. ISO 23903 enables integration of and interoperability between any systems and their components, any domains and their actors, any specifications or products, and any IT-specific view of ISO/IEC 10764. Without following the ISO 23903 model and framework, the integration of and interoperability between specifications and standards are usually not feasible.

### 3.1. Architecture Standards

Regarding the architectural approach, ISO 23903 builds on ISO/IEC/IEEE 42010:2011 Systems and Software Engineering–Architecture Description (ISO/IEC/IEEE 42010 is originally based on ANSI/IEEE 1471-2000 Recommended Practice for Architectural Description of Software-Intensive Systems) [18] and ISO/IEC/IEEE 42020:2019 Software, Systems and Enterprise–Architecture Processes [19]. On that basis, ISO/IEC 10746 Open Distributed Processing [13] has been widely introduced, which is a family of international standards for describing and developing distributed systems and applications. Regarding the system development process, ISO 23903 refers to ISO/IEC 10746 and the Rational Unified Process (RUP) [20]. Another architectural approach, reusing the Reference Model of Open Distributing Processing (RM-ODP), is the HL7 Version 3 Development Framework (HDF), advancing the messaging approach HL7 started with. In the context of HL7, ISO/IEC 7498-1:1994 Information technology-Open Systems Interconnection-Basic Reference Model: The Basic Model, providing the basics for HL7, should be mentioned here as well [21].

Almost all architecture standards focus on the ICT perspective and ignore the importance of real-world communication and cooperation between the domain experts, which is, however, crucial for all ecosystems and especially for the 5P medicine ecosystems. ISO 23903 extended the aforementioned standards like, ISO/IEC 10746, from the business perspective represented by domain experts. Contrary to those standards, ISO 23903 introduced a three-dimensional model with the additional domain perspective dimension to represent multiple domains involved in the ecosystem’s specific use cases and with the additional component composition dimension, thereby reusing the OMG Model Driven Architecture (MDA) hierarchy [22]. The latter starts with the computation-independent model (CIM) or requirement model defining the system in its environment. CIM is transformed into the platform-independent model (PIM), or analysis and design model, defining the system’s architecture. PIM is then transformed into the platform-specific model (PSM) or realization model, defining how the system is built using specific technologies and programming languages. At the end, the code of the system and configuration artifacts are generated [23]. An overview of architecture standards and approaches, including their relation to ISO 23903, is provided in [24]. Table 4 compares the aforementioned data model levels as well as the dimensions of modeling with the model and framework of ISO 23903 and ISO/IEC 10746.

All architecture standards presented so far are business-domain-independent. However, there are also domain-specific architecture standards such as ISO 12967 Health informatics–Service architecture [26].

### 3.2. Knowledge Representation and Knowledge Management Standards

Regarding the business system representation from the perspective and context of the domain experts involved by formally representing their knowledge, we deploy the related domain ontologies. An ontology provides an explicit specification of a conceptualization [27]. It is a collection of terms, relational expressions and associated natural-language definitions in combination with formal theories [28] to represent that knowledge.

Medical/clinical domain terminologies and ontologies for 5P medicine ecosystems are, e.g.,: the Unified Medical Language System (UMLS) [29]; the SNOMED International products Systematized Nomenclature of Medicine–Clinical Terms (SNOMED CT) and Systematized Nomenclature of Medicine Clinical Term Ontology (SCTO) [30]; ISO 25,720 Genomic Sequence Variation Markup Language [31]; Human Phenotype Ontology (HPO) [32]; Infectious Diseases Ontology (IDO) [33]; Epilepsy and Seizures Ontology (EPSO) [34]; Alzheimer’s Disease Ontology (ADO) [35]; the Gene Ontology (GO) [36], and many more. A specific representation of care systems is provided by ISO 13940 Health informatics–System of concepts to support Continuity of care [37]. Its representation style is placed below formal ontologies but above IT systems representation in the enterprise view because of the definition of clinical concepts. An overarching medical ontology, not limited to a specific medical domain but covering the entire health business, has been recently published in [38].

**Figure 3:**
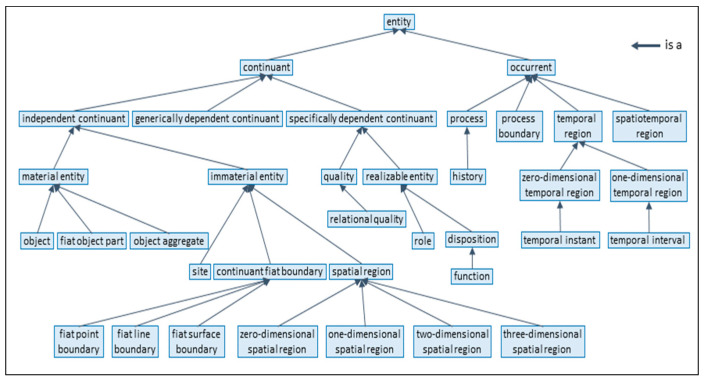
Basic Formal Ontology (BFO) is a hierarchy (after ISO/IEC 21838:2020) [28].

For mapping and matching different ontologies to enable cross-domain communication and collaboration, the ontologies have to be represented or re-engineered, respectively, as formal entities, including their contexts, constraints, and relationships, by using attributes and relations according to ISO/IEC 21838:2021 Information Technology–Top Level Ontologies (TLO) [28] (Figure 3). In case no ontologies are available for representing a specific domain or subdomain, a preliminary ontology can be derived from the TLO base classes.

### 3.3. The Policy Domain

**Figure 4:**
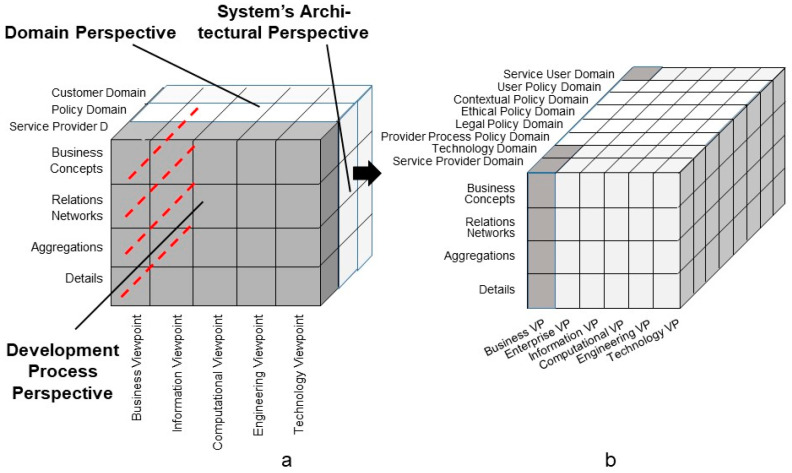
Specialization of the policy domain into sub-policy domains relevant for P5 medicine ecosystems (after [39], changed).

A policy defines a set of legal, regulatory, procedural, ethical, and contextual requirements and obligations for communication and cooperation, including privacy and trustworthiness. That way, controlling the intended behavior of business systems, a policy domain representing policy knowledge, concepts, and relations, is crucial for defining, designing, and running any type of ecosystem. Using the ISO 23903 model and framework, Figure 4 demonstrates the specialization of the policy domain into the sub-policy domains relevant for P5 medicine ecosystems. The user policy domain—sometimes also called personal policy domain or individual’s policy domains—represents the intentions, expectations, wishes, etc., of the individual engaged in the business case, such as a patient.

Examples for a provider process policy domain instance are best practice clinical guidelines. All sub-policy domains must be represented using related ontologies.

**Figure 5:**
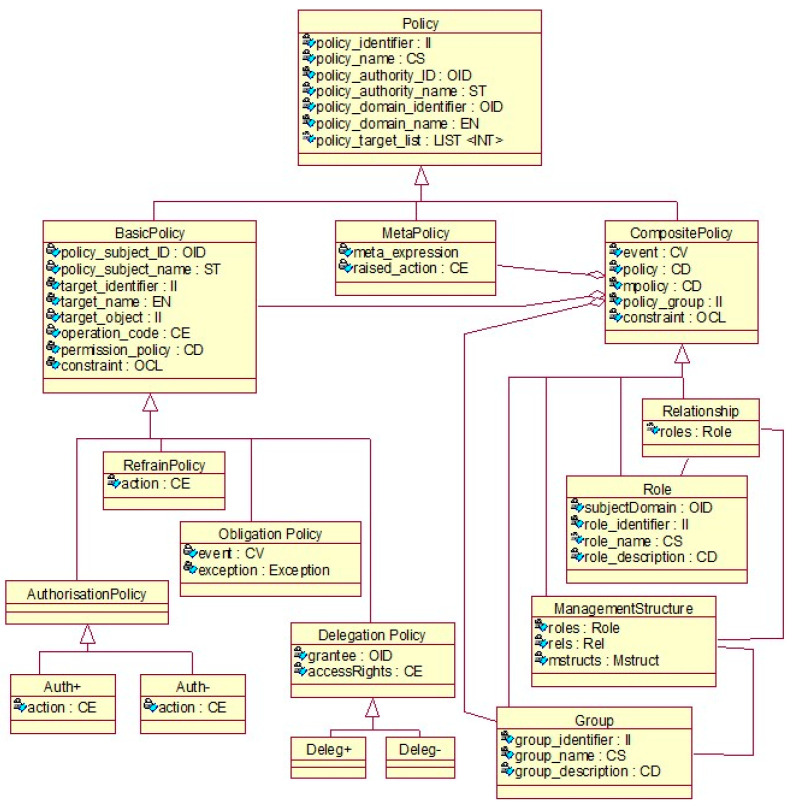
Policy domain components according to ISO 22600-2 [41].

Based on the Ponder Language specification [40], a policy ontology to formalize the rules and constraints controlling the behavior of a business system has been provided by ISO 22600 [41], instantiated for the security and privacy domain (Figure 5).

**Figure 6:**
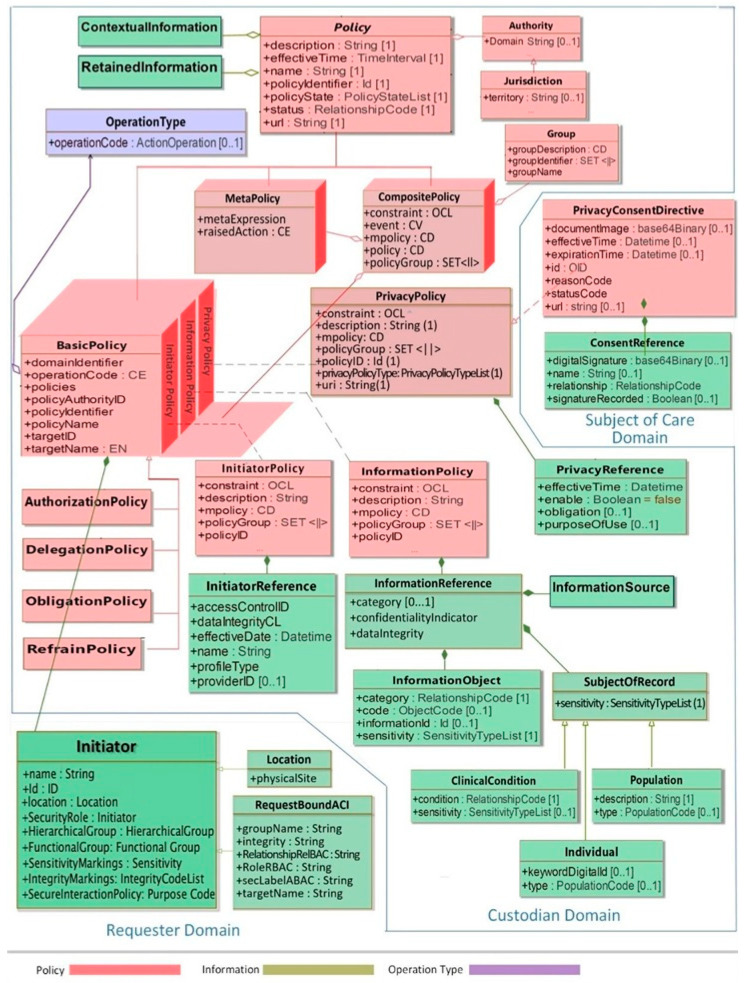
HL7 Privacy and Security Logical Data Model, Release 1, June 2021 [42].

The integration of that policy ontology in an ecosystem for managing security and privacy, using ISO 23903, has been performed in the HL7 Privacy and Security Logical Data Model, Release 1, June 2021 [42] (Figure 6). There are also more ontology-based approaches available [43,44].

For managing ethical and trust aspects of autonomous and intelligent 5P medicine ecosystems, IEEE defined the IEEE 7000 Standard Model Process for Addressing Ethical Concerns during System Design [45] as a framework for specifying ethical issues in IT systems. On that basis, a series of standards with the involvement of the first author have been developed at IEEE. A foundational specification for designing and managing transformed 5PM ecosystems according to the ISO 23903 model and framework is the first global ontological standard for ethically driven robotics and automation systems (ERAS) [46]. More information can be found in [3].

## 4. A Short Overview on Standard Classes and Related Specifications

Table 5, presents for the classes architecture standards; modeling standards; terminology and ontology standards; communication standards; policy, security, and privacy standards; safety standards; and identifier and identification standards some international specifications relevant in the context of P5 medicine ecosystems. Of course, the presented standard types and examples list is not intended to be complete.

## 5. Managing the Modeling and Development Process of 5P Medicine Ecosystems

### 5.1. Representation of 5P Medicine Ecosystems through Standards

**Figure 7:**
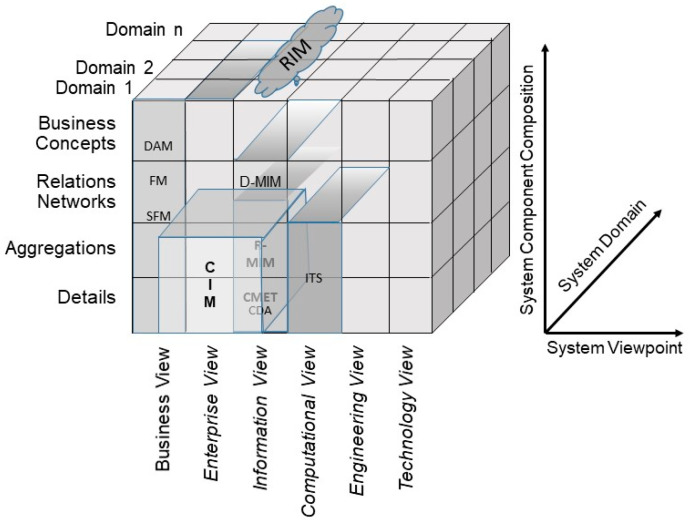
Healthcare-specific specifications representing different ISO 23903 views.

**Figure 8:**
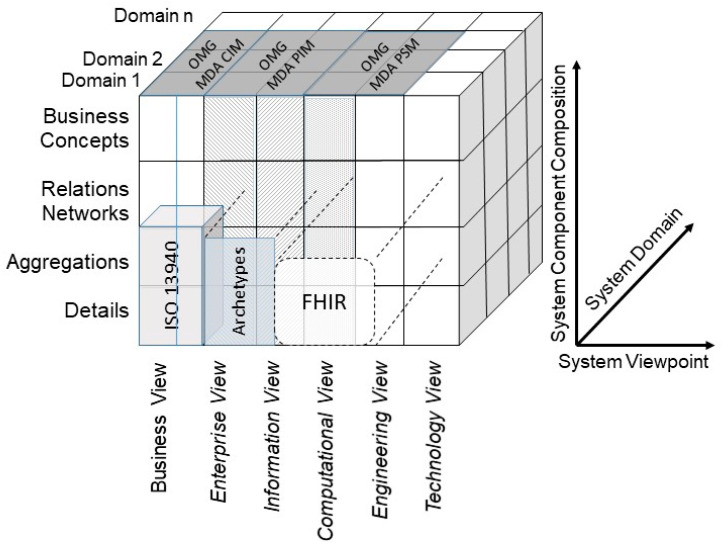
Healthcare-specific specifications representing different ISO 23903 views.

When modeling and developing 5PM ecosystems or managing the integration and interoperability challenge, we are inevitably bound to the good modeling best practices [12] realized by ISO 23903 model and framework [15] by strictly performing a top-down approach. In other words, we cannot jump to a specific viewpoint to correctly interrelate models and artifacts. Instead, we must first solve the mapping between the involved domains represented by domain ontologies to correctly and formally represent the considered multi-disciplinary business system use case. Thereafter, we have to perform the transformation into the ICT-specific views from the enterprise viewpoint through the information view, the computational view down to the engineering viewpoint representing the implementable artifacts. Thereby, we must deploy the related ICT ontologies, from business process modeling through information modeling up to data modeling. While this process, including the representation styles, is clearly specified for the ICT domain perspective by using ISO/IEC 10746 Open Distributed Processing [13] and related specifications, the ontologies and representation styles in health informatics may be healthcare-specific and changing over time. Healthcare-specific standards for representing domain-specific business views are, e.g., the HL7 Domain Analysis Models (DAM) or the ISO or CEN Health Informatics Functional Models (FM) or Services Functional Models (SFM). An example for the first group is the HL7 Composite Security and Privacy Domain Analysis Model (CSP-DAM), meanwhile replaced by the aforementioned HL7 Privacy and Security Logical Data Model, R1 [42]. Examples for the latter group are the HL7 EHR-System Functional Model, R2 (HL7 EHR-S FM), the HL7 PHR-System Functional Model, R2 (HL7 PHR-S FM), or the HL7 Service Functional Models like the HL7 Common Terminology Services 2 Functional Model or the HL7 Version 3 Standard Identification Service R1. Also, the ISO 13940 System of Concepts to Support Continuity of Care [37] must be mentioned here. A newer example for representing health enterprise view components are clinical information models according to ISO 139722 Clinical Information Models [47] or the openEHR [48] and ISO 13606 Electronic Health Record Communication [49,50] archetypes. Thereby, some aspects of the business view as well as the informational representation (information view) are covered. Standards for healthcare-specific information view representations have been established in the HL7 Clinical Document Architecture (HL7 CDA) series [51]. Computational view representation examples are HL7 Implementable Technology Specifications (ITS) but also the globally pushed HL7 Fast Healthcare Interoperability Resources (HL7 FHIR) [52]. Figure 7 and Figure 8 represent the different standards and representation styles in the ISO 23903 Interoperability and Integration Reference Architecture model and framework. Regarding FHIR, starting as an implementable resource as expressed in Figure 8, five levels are meanwhile supported. The highest Level 5 covers knowledge-related aspects such as clinical reasoning, Level 4 covers process-related aspects, Level 3 covers semantic interpretations, Level 2 covers service implementations, and Level 1 covers technical representations.

### 5.2. Integrating Existing Standards in 5P Medicine Ecosystems

After discussing some detail in the modeling and development of 5P medicine ecosystems, we will now address the challenge of mapping/matching or integrating existing specifications and artifacts using the model and framework of the ISO 23903 Interoperability and Integration Reference Architecture. To meet this challenge, we must understand the perspectives, objectives, concepts, contexts, etc., that the designer and developer of the component had in mind. Without that knowledge, which is normally not provided with the specification, any integration, mapping, or matching is not decidable. Therefore, we must re-engineer that missing knowledge. As the aforementioned conditions might change from use case to use case, the provided interoperability and integration outcome are specific to the considered use case or related classes of use cases, and the procedure has to be performed again for any new settings and contexts.

**Figure 9:**
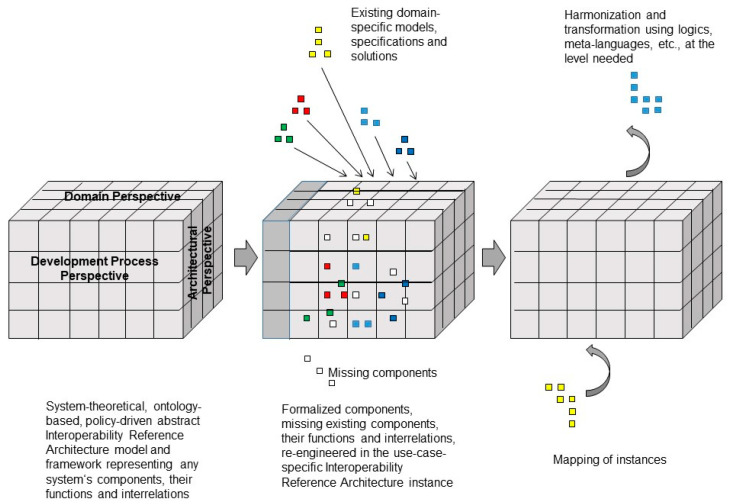
Integration of standards and specifications using the ISO 23903 Reference Architecture model and framework.

In the first step, the components in question must be correctly placed into the ISO 23903 model regarding the domain, the granularity level, and the represented development process viewpoint. Thereafter, the concepts represented by the considered components must be formally modeled in the business view using the corresponding domain ontologies as well as top-level ontologies for interrelating them. The concepts must be completed to correctly and operationally represent the real-world business system and business processes for the use case to be enabled or supported. The resulting business system representation must then be transformed into views according to the development process up to the considered components’ view. This includes a re-engineering of the components and relationships, i.e., classes, attributes, operations, and relations needed to represent the full business use case must be added or modified. Figure 9 represents the described procedure.

**Figure 10:**
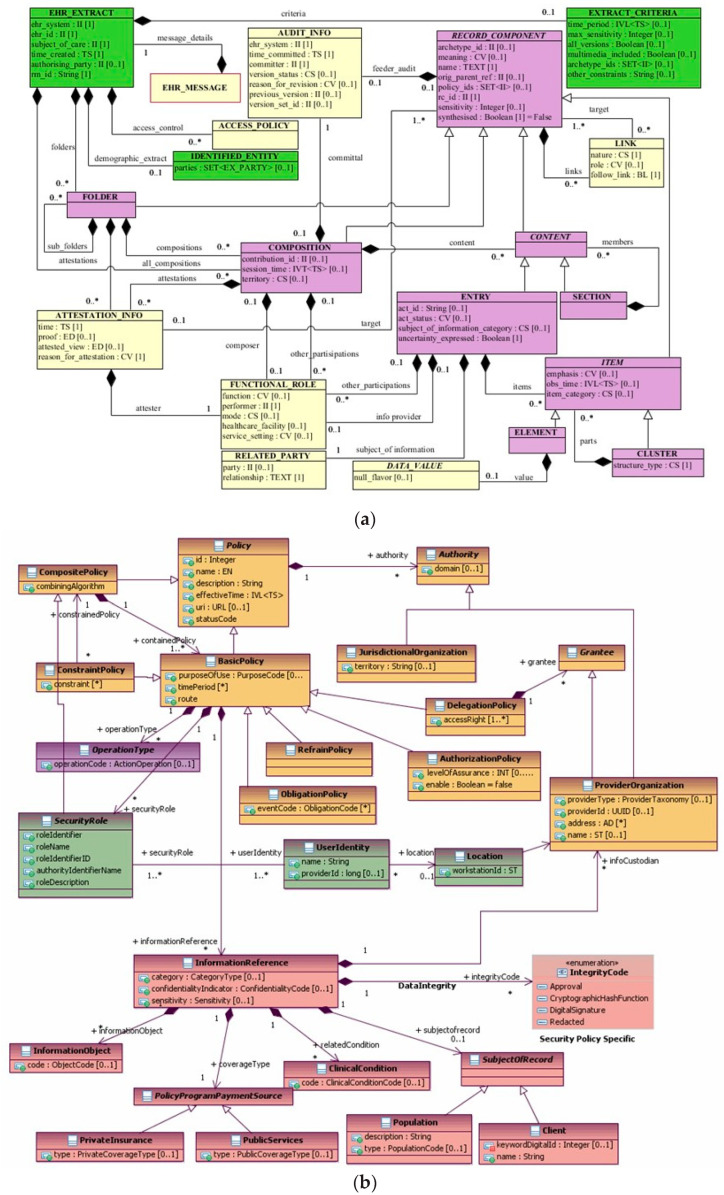

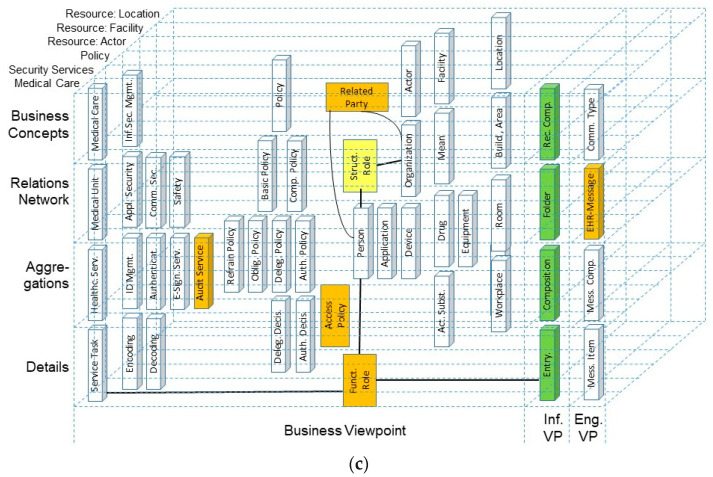
(**a**) ISO 13606-1 Reference Model [49]. (**b**) HL7 V3 Composite Security and Privacy DAM [53]. (**c**) Integrating ISO 13606-1 Reference Model and the HL7 V3 Composite Security and Privacy DAM regarding security and privacy aspects using ISO 23903.

Following, we will exemplify the procedure of integrating and mapping specifications for enabling comprehensive interoperability as presented in ISO 23903:2021. First, we demonstrate the integration of security and privacy aspects specified in different standards, such as ISO 13606-1:2019 Health informatics-Electronic health record communication-Part 1: Reference model [49] (Figure 10a) and the HL7^®^ Composite Security and Privacy Domain Analysis Model [53] (Figure 10b). Reengineering and mapping both standards by using the ISO 23903 model and framework is demonstrated in Figure 10c. That way, the more advanced security and privacy specifications provided by HL7 can be integrated into EHR solutions based on ISO 13606.

**Figure 11:**
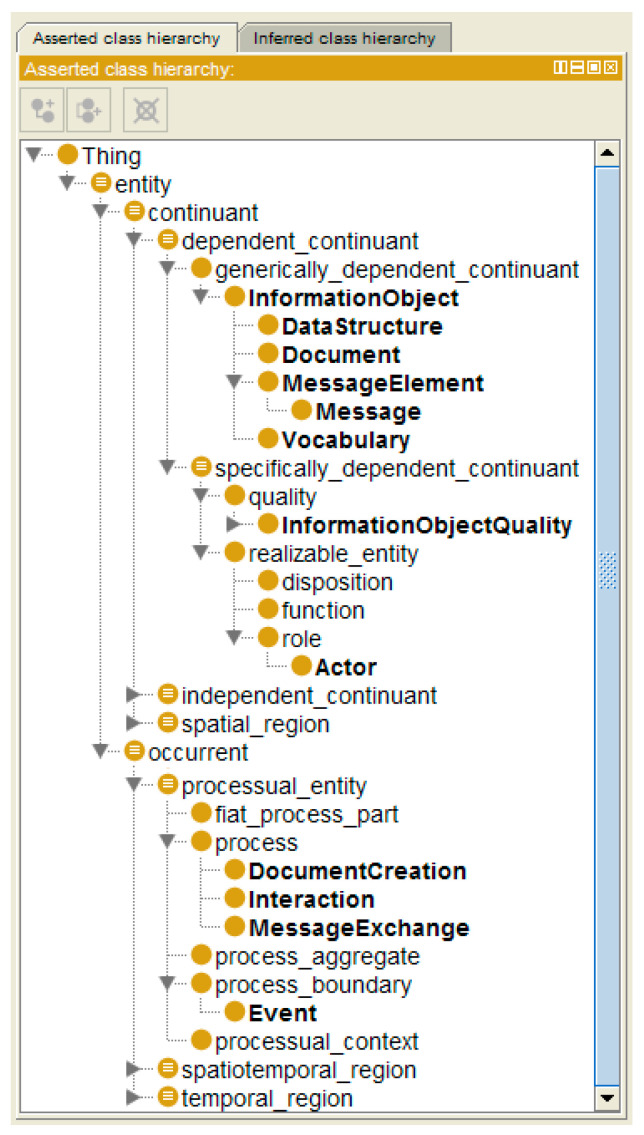
The Communication Standards Ontology presented in Protégé [55].

Another example deals with mapping HL7 V2 and HL7 V3 models and specifications. While HL7 V3 is based on the standardized HL7 Development Framework (HDF) related to ISO/IEC 10746 [13] or the Rational Unified Process (RUP) [20], respectively, HL7 V2 does not have a formal development framework or foundation but has been specified “on the fly” by borrowing from another ASTM standard and simply adjusting it to healthcare needs. Therefore, the HL7 V2 process must first be formally analyzed and represented (re-engineered), following the ISO 23903 principles. Furthermore, it must be ontologically represented using the Communication Standards Ontology (CSO) developed by Frank Oemig in the context of his PhD work [54] (Figure 11). The CSO elements integrated into the aforementioned BFO [28] are presented in bold [55].

**Figure 12:**
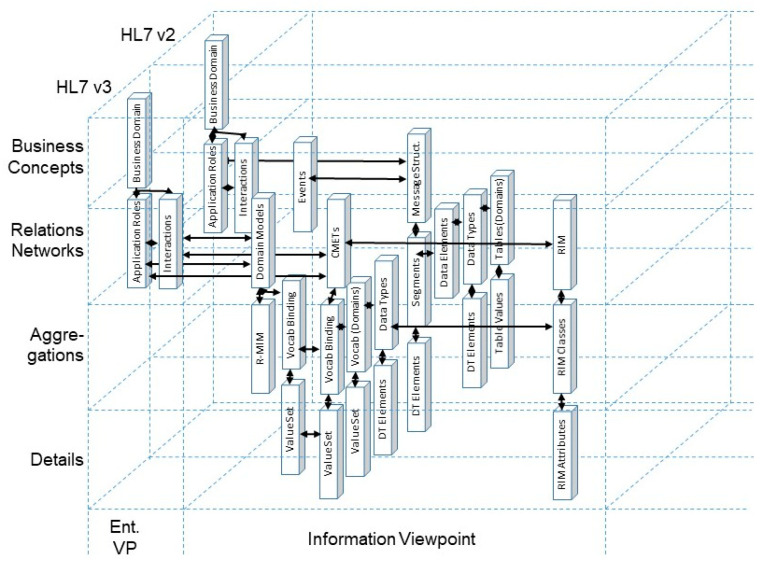
Re-engineering the development process model of HL7 v2^®^ and HL7v3^®^ for integrating the two communication standards [15].

The outcome of re-engineering HL7 V2 and V3 according to ISO 23903 and representing them using the Communication Standards Ontology for mapping the specifications is shown in Figure 12.

**Figure 13:**
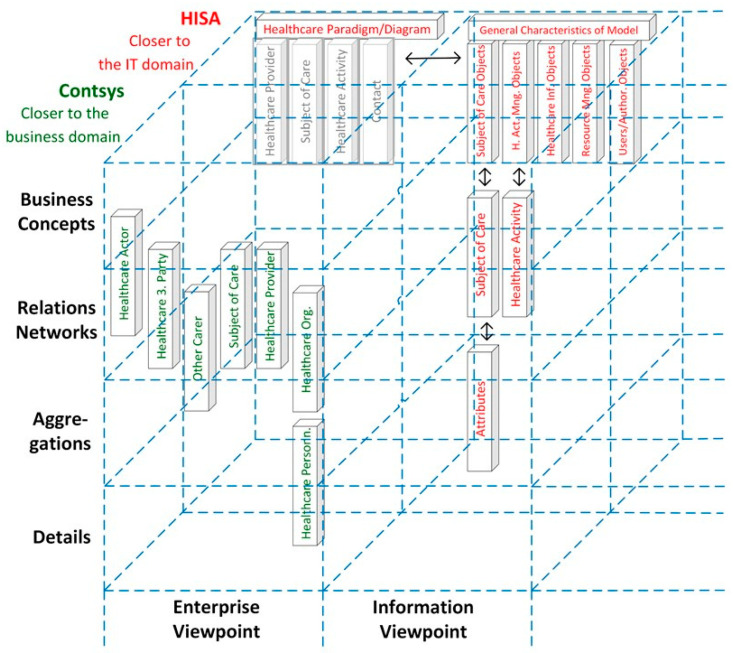
Re-engineering example of selected parts of ISO 12967 (all parts) and ISO 13940.

Another integration example is provided in Figure 13, demonstrating the re-engineering and mapping of the higher-level specifications ISO 12967 Health Informatics Service Architecture [26] and ISO 13940:2015 System of concepts to support continuity of care [37].

## 6. Summary and Conclusions

For designing, implementing, and managing transformed health ecosystems, we cannot simply integrate system components (specifications, standards, and artifacts) from a specific IT system viewpoint perspective, i.e., enterprise concepts representing enterprise knowledge, terms representing information, or data. When this has been completed, e.g., by combining special FHIR resources or mapping specifications from different standards just based on representational characteristics in one viewpoint, such as terminologies, naming conventions, etc., performed in some ISO/TC 215 projects, the outcome is incompatible, inconsistent, and therefore unsuitable. Instead, we must understand and formally represent the ecosystem using a system-oriented, architecture-centric, ontology-based, and policy-controlled approach, acknowledging the limitations of the data focus for specifying ecosystems.

Building on many years of work in health care with responsibilities for designing, implementing, and using related information systems, including necessary infrastructure services for interoperability, security, privacy, etc., but also for advancing health with telemedicine, pHealth, and eHealth, the first author developed the cross-domain and technology-independent interoperability and integration reference model and framework, domain-specifically supported by some of the co-authors. The basis for the approach, the Generic Component Model (GCM), dates from the early nineties of the last century and has successfully evolved over time. Meanwhile, the Health Informatics TCs of ISO and CEN, as well as other SDOs, mandated the use of ISO 23903 for any project covering multiple aspects or domains, as well as for realizing integration and interoperability between system components, including specifications and artifacts. The described limitations of constraint representation language result in the need to advance from data sharing interoperability to knowledge sharing interoperability in dynamic and complex intercultural, interdisciplinary, and inter-jurisdictional environments. Additionally, this was the driving factor for replacing the EU Data Protection Directive [56] by the EU General Data Protection Regulation (GDPR) [57], i.e., advancing from a privacy-related data classification towards the detailed consideration of processes and contexts of creating, collecting, using, and sharing personally identifiable information (PII) [58]. Projects such as the European Health Data Space [59] are therefore more than questionable (see, e.g., [9]). The nature of 5P medicine requires a concept- and context-based approach, including accompanying privacy and ethical aspects.

The presented approach enables comprehensive and consistent integration of and interoperability between domains, systems, related actors, specifications, standards, and solutions without limiting the used languages and methodologies. Thereby, it advances interoperability beyond the still dominant syntactic and semantic level towards knowledge sharing at the business concept level (agreed cooperation), knowledge sharing at the domain level (cross-domain cooperation), and even knowledge sharing in the individual context of education, experiences, and skills (moderated end-user collaboration).

## Figures and Tables

**Table 1:** Organizational, methodological, technological, and standardization paradigm changes in transformed health and social care ecosystems.

Care Type	Organization, Service Provision	Actors	Services	Target	Way of Practicing	Justification	Representation Style	Electronic Comm./ Co-op.	Standard
Phenomenological medicine	Organization-centered, local services	Regulated professionals	Domain-specific general services	Humanity	Observation	Pattern recognition	Data	Local data repository; inside the unit	Data standards
Evidence-based medicine	Organization-centered, local services	Regulated professionals	Domain-specific, group-specific services	Disease-specific defined group	Observation with objective evaluation	Statistical justification, group-specific treatment outcome	Information	Central data repositories	Information standards
Person-centered medicine	Cross-organizational local services	Regulated professionals	Multiple domains’ services	Individual	Managed care	Process mgmt., best medical practice guidelines	Agreed terminology, DMP Best Practice Guidelines	Cross-organizational business process	Terminology standards; process standards
Personalized medicine	Distributed local and remote services	Regulated and non-regulated professionals, laymen, technical systems	Multiple domains’ services—telemedicine	Individual’s personal disposition	Considering the pathology of disease	Clinically justified individual status and context	Disciplinary concepts in situational context	Knowledge management	Domain ontology standards
5P medicine	Distributed cross-domain services, smart healthcare	Regulated and non-regulated professionals, laymen, technical systems	Cross-domain services—consumerism, telemedicine	Individual in personal, environmental, social, occupational, and behavioral contexts	Understanding the pathology of disease	Scientifically justified individual status and context	Multidisciplinary concepts in comprehensive context	Knowledge space management	Multiple ontologies guided via top-level ontology standards
Ubiquitous personal health and social care	Ubiquitous services	Regulated and non-regulated professionals, laymen, technical systems	Integrated services—consumerism, ubiquitous medicine	Individual under comprehensive focus		Dynamically and scientifically justified individual status			

**Table 2:** 5P medicine objectives, characteristics, and methodologies/technologies to meet objectives (after [8], changed).

Objective	Characteristics	Methodologies/Technologies
Provision of health services everywhere, anytime	Openness Distribution Mobility Pervasiveness Ubiquity	Wearable and implantable sensors and actuators Pervasive sensor, actuator, and network connectivity Embedded intelligence Context awareness
Individualization of the system according to status, context, needs, expectations, wishes, environments, etc., of the subject of care	Flexibility Scalability Cognition Affect and behavior Autonomy Adaptability Self-organization Subject of care involvement Subject of care centration	Personal and environmental data integration and analytics Service integration Context awareness Knowledge integration Process and decision intelligence Presentation layer for all actors
Integration of different actors from different disciplines/domains (incl. the participation/empowerment of the subject of care), using their own languages, methodologies, terminologies, ontologies, thereby meeting any behavioral aspects, rules, and regulations	Architectural framework End-user interoperability Management and harmonization of multiple domains including policy domains	Advanced systems architecture Terminology and ontology management and harmonization Knowledge harmonization Language transformation/translation
Usability and acceptability of 5P Medicine solutions	Preparedness of the individual subject of care—security, privacy, trust, and ethics framework Consumerization Subject of care empowerment Subject of care as manager Information-based assessment and selection of services, service quality, and safety as well as trustworthiness Lifestyle improvement and Ambient Assisted Living (AAL) services	Tool-based ontology management Individual terminologies Individual ontologies Tool-based enhancement of individual knowledge and skills Human-centered design of solutions User experience evaluation Individual, context-sensitive privacy agreements Trust calculation services

**Table 3:** Representation tools for the different ISO 10746 viewpoints [9].

Viewpoint	Language/Grammar	Representation Level
Business View	Domain-specific and/or natural languages	
Enterprise View	Business Process Modeling Language (BPML)	Very high
Information View	Unified Modeling Language (UML)	High level
Computational View	Object Constraint Language (OCL)	Logical level
Engineering View	Programming languages with different levels of grammar	Physical level
Technology View		

**Table 4:** Comparing data model levels and dimensions of modeling with ISO 23903 and ISO 10746 (after [25], changed).

Data Model Level	Modeling Actors	Model Scope	Dimension of Modeling	Interop. Reference Architecture	Examples		
**Very-high-level data model**	Business domain stake-holders	Scope, requirements, and related basic concepts of business case	Knowledge space	Business View	OMG CIM		ISO 23903 Interoperability and Integration Reference Architecture
**High-level data model**	Business domain stake-holders	Relevant information and representation and relationships of basic concepts	Knowledge	Enterprise View	OMG PIM, HL7 DCM, CSO	ISO 10746 ODP-RM	
**Logical data model**	Data modelers and analysts	Layout and types of data and object relationships	Information	Information View	OMG PSM, HL7 V3 (CMETs), HL7 CIMI, openEHR Archetypes, FHIM		
				Computational View	HL7 FHIR		
**Physical data model**	Data modelers and developers	Implementation-related and platform-specific aspects	Data	Engineering View			

**Table 5:** Standard classifications and related international standard examples.

Standards Classification	Examples
Architecture standards	HL7 Version 2.x/3, OMG CORBA, OMG MDA, ISO 12967 Health informatics–Service architecture (HISA), ISO 7498-2:1989, Information processing systems—Open Systems Interconnection—Basic Reference Model—Part 2: Security Architecture, ISO 13407:1999 Human-centred design processes for interactive systems
Modelling standards	OMG Unified Modeling Language (UML), ISO/IEC 19505-2:2012 Unified Modeling Language (UML), CEN 15300 CEN Report: Framework for formal modelling of healthcare security policies
Terminology and ontology standards	UMLS, Systematized Nomenclature of Medicine Clinical Terms (SNOMED CT), Systematized Nomenclature of Medicine Clinical Term Ontology (SCTO), ISO 25720 Genomic sequence variation markup language, ISO/IEC 2382-8:1998 Information technology—Vocabulary—Part 8: Security, CEN-ENV 13608-1:2000 Health informatics—Security for healthcare communication—Part 1: Concepts and terminology, ISO 13940:2015 Health informatics—System of concepts to support continuity of care, Logical Observation Identifiers Names and Codes (LOINC), Unified Code for Units of Measure (UCUM)
Communication standards	ISO/IEC 7498-1:1994 Information technology—Open Systems Interconnection—Basic Reference Model: The Basic Model, HL7 V2.x/3, HL7 FHIR (Fast Healthcare Interoperability Resource), X12 EDI, UN EDIFACT, H.PRIM, xDT, Odette FTP, CEN 13606 Electronic healthcare record communication, ISO/IEEE 11073 Health informatics—Point-of-care medical device communication, ISO 17113 Health informatics–Exchange of information between healthcare information systems–Development of messages, CDISC and DICOM specifications, Classification Markup Language (ClaML), EN ISO 27269:2022 Health informatics-International patient summary (ISO 27269:2021)
Policy, security, and privacy standards	ISO/IEC 2700 Information security management, ISO 22600:2014 Health informatics–Privilege management and access control, ISO 17090 Public key infrastructure, ETSI TS 101733 Electronic Signature Formats, ASTM E1987-98 Standard guide for individual rights regarding health information, CEN 13608 Security for healthcare communication, CEN 13729 Secure user identification-Strong authentication using microprocessor cards, ISO 25237:2017 Health informatics—Pseudonymization, ISO/IEC PDTS Pseudonymisation Practices for the Protection of Personal Health Information and Health Related Services, ISO/IEC 27018:2019 Information technology—Security techniques—Code of practice for protection of personally identifiable information (PII) in public clouds acting as PII processors, ISO/IEC 29151:2017 Information technology—Security techniques—Code of practice for personally identifiable information protection, ISO 21298:2017 Health informatics–Functional and structural roles, ISO/IEC 9594-8:2008, Information technology—Open Systems Interconnection—The Directory: Public-key and attribute certificate frameworks, ISO/IEC 9798-3:1998, Information technology—Security techniques—Entity authentication—Part 3: Mechanisms using digital signature techniques, ISO/IEC 10181-1:1996, Information technology—Open Systems Interconnection—Security frameworks for open systems: Overview, ISO/TS 17090-1:2013 Health informatics—Public key infrastructure—Part 1: Overview of digital certificate services, ENV 13729:1999, Health informatics—Secure user identification for healthcare strong authentication using microprocessor cards, ISO 21091:2013 Health informatics—Directory services for healthcare providers, subjects of care and other entities, ISO/IEC 15408-1:2009 Information technology—Security techniques—Evaluation criteria for IT security—Part 1: Introduction and general model
Safety standards	CEN 13694 CEN Report: Safety and security related software quality standards for healthcare, ISO/DTS 25238 Classification of Safety Risks, IEC 82304-1 Health Software–Part 1: General requirements for product safety. IEC 82304-2 Health Software–Part 2: Health and wellness apps–Quality and reliability
Identifier and identification standards	LOINC, ASTM E1714-00 Standard guide for properties of a Universal Healthcare Identifier
Document standards	HL7 V3/CDA (Clinical Document Architecture), DICOM SR (Structured Reporting), HL7 FHIR Bundle+Composition
Data representation (visualization) standards	HTML, PDF, PDF/A, MS Word, ClaML
Encoding standards	XML, JSON, ASN.1, ER7, xDT
Character representation standards	ASCII, EBCDIC, Unicode

## Data Availability

The original contributions presented in the study are included in the article. Further inquiries can be directed to the corresponding author.
